# Aromatherapy for managing menopausal symptoms: A protocol for systematic review and meta-analysis

**DOI:** 10.1097/MD.0000000000009792

**Published:** 2018-02-09

**Authors:** Jiae Choi, Hye Won Lee, Ju Ah Lee, Hyun-Ja Lim, Myeong Soo Lee

**Affiliations:** a Clinical Research Division, Korea Institute of Oriental Medicine; b KM Convergence Research Division, Korea Institute of Oriental Medicine, Daejeon; c Department of Korean Internal Medicine, College of Korean Medicine, Gacheon University, Incheon; d Deportment of Nursing, Chodang University, Muan; e Present address: Healthy Life Management Team, Korea Health Promotion Institute, Seoul, Republic of Korea.

**Keywords:** aromatherapy, essential oil, menopausal symptoms, randomized controlled trials, systematic review

## Abstract

**Background:**

Aromatherapy is often used as a complementary therapy for women's health. This systematic review aims to evaluate the therapeutic effects of aromatherapy as a management for menopausal symptoms.

**Methods:**

Eleven electronic databases will be searched from inception to February 2018. Randomized controlled trials that evaluated any type of aromatherapy against any type of control in individuals with menopausal symptoms will be eligible. The methodological quality will be assessed using the Cochrane risk of bias tool. Two authors will independently assess each study for eligibility and risk of bias and to extract data.

**Results:**

This study will provide a high quality synthesis of current evidence of aromatherapy for menopausal symptoms measured with Menopause Rating Scale, the Kupperman Index, the Greene Climacteric Scale, or other validated questionnaires.

**Conclusions:**

The conclusion of our systematic review will provide evidence to judge whether aromatherapy is an effective intervention for patient with menopausal women.

**Ethics and dissemination:**

Ethical approval will not be required, given that this protocol is for a systematic review. The systematic review will be published in a peer-reviewed journal. The review will also be disseminated electronically and in print.

**Systematic review registration:**

PROSPERO CRD42017079191.

## 1 Introduction

Menopause induces several uncomfortable symptoms, including hot flashes, depression, vaginal dryness, low libido, osteoporosis, fatigue, sleep disturbances, palpitations, emotional imbalance, etc.^[1]^ The symptoms of menopause can be effectively treated with hormone replacement therapy (HRT), including estrogens combined with progestagens or estrogens alone.^[2]^ However, many women are concerned about the much-publicized risks of this therapy and, therefore, look for alternatives.^[3–6]^ The use of phytoestrogens such as aromatherapy may relieve menopausal symptoms and improve lipid profiles in postmenopausal women.^[7]^

Aromatherapy uses essential oils extracted from herbs, flowers, and other plants to improve physical, emotional, and spiritual well-being and to treat various diseases through inhaling, massage, or bath treatment.^[7]^ Many clinical studies on aromatherapy have shown that it is beneficial for reducing stress and pain, enhances alertness and feelings of relaxation, and reduces anxiety by stimulating endorphin production.^[7–11]^

A recent systematic review included 5 trials (2 randomized clinical trials [RCTs] and 3 controlled clinical trials) on the effects of aromatherapy on menopausal symptoms.^[12]^ Its findings suggested that aromatherapy is effective for managing menopausal symptoms. However, the review lacked a comprehensive literature search and was outdated. Hence, the aim of the current systematic review is to summarize and critically evaluate the evidence for or against the effectiveness of aromatherapy as management of menopausal symptoms.

## 2 Methods

### 2.1 Registration

The protocol of this systematic review has been registered on PROSPERO 2017 CRD42017079191) (Available from: http://www.crd.york.ac.uk/PROSPERO/display_record.php?ID=CRD42017079191). This systematic review protocol was reported using the Preferred Reporting Items for Systematic Reviews and Meta-Analyses (PRISMA-P) statement guidelines.^[13]^

### 2.2 Search methods for identifying the studies

#### 2.2.1 Electronic sources

The following electronic databases will be searched for studies published from their dates of inception to February 2018: AMED (EBSCO), CINAHL (EBSCO), EMBASE (EBSCO), MEDLINE (PubMed), and the Cochrane Central Register of Controlled Trials (CENTRAL), as well as Korea Med, Oriental Medicine Advanced Search Integrated System (OASIS), DBPIA, the Korean Medical Database (KM base), the Research Information Service System (RISS), and the Korean Studies Information Services System (KISS). In addition, the reference lists of potentially eligible articles will be searched manually to identify additional relevant reports. In addition, the reference lists of all identified articles will be further searched for potentially relevant papers. Hard copies of all the articles will be obtained and read in full. The search strategy will be used with modifications of this for each database.

#### 2.2.2 Search strategies

The searches will be conducted using the medical subject headings (MeSHs) “aromatherapy OR essential oil” AND “menopause OR climacteric OR menopausal.”

### 2.3 Eligibility criteria

#### 2.3.1 Types of studies

All the prospective RCTs identifying the therapeutic effects of aromatherapy compared with no treatment, placebo or conventional medication will be included.

#### 2.3.2 Types of participants

We will include menopausal women: women who are going through the menopausal transition or women who are postmenopausal.

#### 2.3.3 Types of interventions

The review will include all trials of any duration that investigated the effects of any type of aromatherapy, including aromatherapy administered via massage or the inhaled or oral routes, regardless of how the therapy was dosed, prepared, or processed. We will compare all types of control interventions, including placebo treatments, conventional medicines, standard care methods, and no treatments. Studies comparing 2 types of aromatherapy will be excluded from the review.

#### 2.3.4 Outcome measures

##### 2.3.4.1 Primary outcomes

Menopausal symptoms (hot flashes, night sweats, vaginal dryness, etc.) measured with Menopause Rating Scale, the Kupperman Index, the Greene Climacteric Scale, or other validated questionnaires

Female sexual function measured with the validated questionnaires including the Female Sexual Function Index, the Brief Index of Sexual Functioning for Women, Sexual Functioning Questionnaire, etc.

##### 2.3.4.2 Secondary outcomes

- Adverse effects
- General well-being or quality of life

### 2.4 Data collection and analysis

#### 2.4.1 Study selection

The data screening and selection process will be performed independently by 2 authors (JC and HWL) and will be verified by the third author (JAL). When disagreements on the selection are not resolved through discussions, the arbiter (MSL) will decide. No language restrictions will be imposed. Hard copies of all the articles will be obtained and read in full. Details of the selection process are shown in the PRISMA flow diagram.

#### 2.4.2 Data extraction and management

All the articles will be read by 3 independent reviewers, who will extract the data from the articles according to predefined criteria. The extracted data will include specific details about the authors, years of publication, study designs, sample sizes, interventions (regimens), controls (regimens), main outcome measures, adverse effects, and authors’ conclusions. The extracted data will be tabulated for further analysis.

#### 2.4.3 Risk of biased assessment

We will independently assess bias in the included studies according to the criteria from the Cochrane Handbook version 5.1.0, which includes random sequence generation, allocation concealment, blinding of participants and personnel, blinding of outcome assessment, incomplete outcome data, selective reporting, and other sources of bias.^[14]^ The quality of each trial will be categorized into a low, unclear, or high risk of bias. We will resolve any differences in opinion through discussion or consultation with the third author. The overall quality of this systematic review and meta-analysis will be summarized and evaluated with GRADEpro software (http://www.gradepro.org).

#### 2.4.4 Measures of the treatment effect

The differences between the intervention and control groups will be assessed. For the continuous data, we will use the mean difference (MD) with 95% confidence intervals (CIs) to measure the treatment effect. We will convert other forms of data into MDs. In the case of outcome variables with different scales, we will use the standard MD with 95% CIs. For dichotomous data, we will present the treatment effect as a relative risk (RR) with 95% CIs to assess the effect size of each included study. We will convert other binary data into an RR value.

All of the statistical analyses will be conducted using Cochrane Collaboration's software program, Review Manager (RevMan), Version 5.3.5 for Windows (Copenhagen, the Nordic Cochrane Centre, the Cochrane Collaboration 2014). For studies with insufficient information, we will contact the corresponding authors to acquire and verify the data when possible. Chi-squared and I-squared tests will be used to evaluate the heterogeneity of the included studies. Unless excessive statistical heterogeneity is present, we will then pool the data across studies for a meta-analysis using a random-effects or fixed-effects model.

#### 2.4.5 Unit of analysis issues

For cross-over trials, data from the first treatment period will be used. For trials in which more than 1 control group was assessed, the primary analysis will combine the data from each control group. Subgroup analyses of the control groups will also be performed. Each patient will be counted only once in the analysis.

#### 2.4.6 Dealing with missing data

Intention-to-treat analyses that include all of the populations will be performed. For populations with missing outcome data, a carry-forward of the last observed response will be used. If missing data are detected, we will request any missing or incomplete information from the original source investigators.

#### 2.4.7 Assessment of heterogeneity

If a meta-analysis is possible, we will use the I^2^ statistic to quantify the inconsistencies among the included studies. According to the guidance given in the Cochrane Handbook for Systematic Reviews of Interventions, an I^2^ value of 50% will be the cut-off point for meaningful heterogeneity. If heterogeneity in the meta-analyses is observed, we will conduct a subgroup analysis to explore the possible causes.^[15]^ Subgroup analyses based on different control interventions, types of control, types of condition, and types of study design will be conducted. A sensitivity analysis will be performed to evaluate the robustness of the meta-analysis results.

#### 2.4.8 Assessment of reporting biases

If a sufficient number of the included studies (at least 10 trials) are available, we will use funnel plots to detect reporting biases.^[16]^ If the funnel plot asymmetry is not the same as the publication bias, we will attempt to distinguish the possible reasons for the asymmetry, such as small-study effects, poor methodological quality, and true heterogeneity in the included studies.

## 3 Discussion

This systematic review may provide a detailed summary of the current evidence related to the effectiveness of aromatherapy, as well as useful information on the acceptability and applicability in the field of complementary and alternative medicine research for both practitioners and patients. The findings of this review will be disseminated widely through peer-reviewed publications and conference presentations.
